# Electroacupuncture for slow flow/no-reflow phenomenon in patients with acute myocardial infarction undergoing percutaneous coronary intervention: protocol for a pilot randomized controlled trial

**DOI:** 10.3389/fcvm.2024.1401269

**Published:** 2024-06-18

**Authors:** Yanbin Peng, Xuqiang Wei, Feng Wu, Min Fan, Ke Wang, Jia Zhou

**Affiliations:** ^1^ Acupuncture Anesthesia Clinical Research Institute, Yueyang Hospital of Integrated Traditional Chinese and Western Medicine, Shanghai University of Traditional Chinese Medicine, Shanghai, China; ^2^ Department of Cardiovascular Medicine, Yueyang Hospital of Integrated Traditional Chinese and Western Medicine, Shanghai University of Traditional Chinese Medicine, Shanghai, China

**Keywords:** electroacupuncture, acute myocardial infarction, slow flow/no-reflow, protocol, randomized controlled trial (RCT) including age, gender, BMI, smoking history

## Abstract

**Background:**

Slow flow/no-reflow (SF-NR) during percutaneous coronary intervention (PCI) is associated with poor prognosis of patients with acute myocardial infarction (AMI). Currently, effective treatment is not available for SF-NR. Electroacupuncture (EA) has shown significant efficacy as an adjuvant therapy for many cardiovascular diseases by improving microcirculation and reducing ischemia-reperfusion injury. However, its effects on SF-NR in the AMI patients during PCI are not clear. This pilot trial aims to determine the efficacy of intraoperative EA in alleviating SF-NR in AMI patients undergoing PCI.

**Methods:**

This prospective, single-center, randomized controlled, pilot trial will recruit 60 AMI patients scheduled for PCI at the Yueyang Hospital of Integrated Traditional Chinese and Western Medicine, China. The patients will be randomized in a 1:1 ratio into the EA or the control groups. Patients in the control group will undergo standard PCI. Patients in the EA group will undergo intraoperative electroacupuncture while undergoing standard PCI. Incidence of SF-NR is the primary outcome for this study. This study will also assess secondary outcomes including cardiac biomarkers, inflammatory biomarkers, pain and anxiety scores, electrocardiography parameters, traditional Chinese medicine (TCM) symptom score, and major adverse cardiovascular and cerebrovascular events (MACCE). All the included patients will undergo laboratory tests including routine blood tests, levels of electrolytes, as well as liver and renal function tests. Patients will be followed up for 1 month after the procedure.

**Discussion:**

This pilot trial will provide evidence for the potential benefits of intraoperative EA in improving microvascular perfusion and preventing or alleviating SF-NR during PCI in patients with AMI. If proven effective, intraoperative EA will provide a new and effective strategy against SF-NR and provide evidence for subsequent multicenter trials.

**Clinical Trial Registration:**

ClinicalTrials.gov, identifier (ChiCTR2300072265). Registered on 8 June 2023.

## 1 Introduction

Acute myocardial infarction (AMI) is a life-threatening condition caused by sudden coronary artery occlusion because of plaque rupture or thrombosis. Therefore, timely reopening of the infarct-related artery is necessary, for salvaging the jeopardized myocardium and restoring the blood flow (1). Percutaneous coronary intervention (PCI) is the primary treatment method for relieving the occluded coronary artery and is associated with minimal invasiveness and rapid recovery (2–4). Despite successful recanalization, 41% of the cases report insufficient blood flow that can lead to a severe complication called as slow flow/no reflow (SF-NR) (5, 6). Patients with SF-NR can develop severe complications, such as like ventricular arrhythmias, heart failure, and sudden death, all of which result in poor prognosis and negate the benefits of PCI in the AMI patients (7–9). SF-NR is associated with a five-fold increase in risk of myocardial infarction and a four-fold increase in the mortality rate (10).

The mechanisms causing SF-NR include microvascular embolization, *in situ* thrombosis, endothelial cell damage, microvascular spasms, and inflammation (11–15). Currently, there are no specific universal class I treatment strategies for alleviating SF-NR. Patients with SF-NR are treated with drugs such as vasodilators and glycoprotein IIb/IIIa antagonists, as well as mechanical treatments, including thrombus aspiration (16, 17). However, there is no definitive evidence that these treatments specifically target and alleviate SF-NR. These treatment methods often target only one causative factor and result in inconsistent and unstable effects. Moreover, most of the treatments are associated with significant adverse effects. For example, excessive use of tirofiban is associated with elevated bleeding risk (18), whereas improper thrombus aspiration causes complications such as thrombus dislocation (19). Therefore, there is an urgent need to discover safe and more effective treatment strategies for alleviating SF-NR.

Acupuncture demonstrates various biological effects, including autonomic nervous system regulation, anti-inflammatory properties, enhanced microcirculation, antioxidant activity, reduced lipid levels, and perioperative pain relief (20, 21). Therefore, it may be a promising strategy for addressing coronary microvascular dysfunction, injury, and obstruction following AMI, such as SF-NR. Firstly, it modulates sympathetic and parasympathetic balance, thereby attenuating sympathetic overactivity and enhancing parasympathetic tone, which may contribute to vasodilation and improved coronary microcirculation (22, 23). Secondly, acupuncture exhibits robust anti-inflammatory properties, as evidenced by its ability to downregulate pro-inflammatory cytokines and inhibit the activation of nuclear factor-kappa B (NF-κB), thus mitigating the inflammatory cascade implicated in microvascular injury post-MI (24). Thirdly, acupuncture exerts antioxidant effects by bolstering endogenous antioxidant defenses and scavenging reactive oxygen species, thereby mitigating oxidative stress-induced damage to endothelial cells and preserving microvascular integrity. Notably, EA has been shown to modulate endothelial function, promoting endothelial nitric oxide synthase (eNOS) activity and nitric oxide (NO) production, pivotal mediators of vasodilation and endothelial homeostasis, thereby counteracting endothelial dysfunction associated with MI-induced microvascular impairment (25, 26). Therefore, acupuncture may potentially prevent SF-NR during PCI by reducing endothelial cell damage. Fourthly, acupuncture holds promise in mitigating microvascular spasm and obstruction by virtue of its vasodilatory effects and modulation of vascular smooth muscle tone (27). This is further corroborated by emerging evidence suggesting that acupuncture may regulate the renin-angiotensin-aldosterone system (RAAS), a key regulator of vascular tone and remodeling, thereby conferring vasoprotective effects and attenuating microvascular dysfunction post-MI (28). Furthermore, acupuncture has been shown to stimulate the release of endogenous opioids and neuropeptides, including β-endorphin and substance P, which contribute to pain relief and myocardial protection (29, 30). Additionally, PCI primarily targets large and medium-sized coronary vessels with stenosis but is not effective in treating thin or small-diameter vessels (31). Acupuncture addresses this limitation as an economical and non-invasive option for the treatment of blood flow issues in smaller blood vessels or complex or risky needle insertion situations. Acupuncture is also a viable option for patients seeking to avoid the side effects of drugs (32). Moreover, acupuncture is one of the earliest therapies for emergency treatment in traditional Chinese medicine (TCM) according to ancient classics. The clinical spectrum of acupuncture includes 16 categories with 461 types of human diseases, of which 26% were acute diseases (33). Acupuncture, particularly electroacupuncture (EA), shows significant clinical benefits. EA is a safe and effective intervention that is easier to standardize, more controllable than manual acupuncture, and provides prolonged stimulation, precise intensity control, and enhanced acupuncture sensation (34, 35). Therefore, combinatorial therapy with EA and PCI is a promising strategy for enhancing clinical outcomes, optimizing coronary blood flow, and improving patient prognosis.

Previous reports have suggested that acupuncture may be effective in preventing or alleviating SF-NR. However, it is not known whether electroacupuncture is effective in preventing or alleviating SF-NR in AMI patients undergo PCI, and high-quality randomized-controlled clinical trials (RCTs) are urgently necessary to investigate its efficacy and safety. Therefore, in this pilot RCT, we aim to investigate the potential benefits of intraoperative EA in reducing SF-NR during PCI in the AMI patients. The findings of this study are expected to lay the foundation for subsequent multicenter trials to establish the clinical safety and efficacy of intraoperative electroacupuncture as a novel strategy for managing SF-NR in AMI patients during PCI.

## 2 Methods and analysis

### 2.1 Study design

This pilot trial will adopt a single-center, blinded, randomized controlled design to determine the efficacy of intraoperative EA in preventing or reducing the incidence of SF-NR in patients with AMI undergoing PCI with standard PCI as control. The trial will follow the Chinese AMI management guidelines and the Declaration of Helsinki. This study has been approved by the Ethics Committee [Approval No. 2023-090-01]. All the study participants will be screened according to the inclusion and exclusion criteria. Finally, sixty eligible patients will be recruited and randomly assigned in a 1:1 ratio to either the EA group or the control group after obtaining informed consent. Patients in the acupuncture group will receive electroacupuncture during the standard PCI procedure, whereas patients in the control group will only receive the standard PCI procedure. The trial period will include the treatment duration from the time of hospital admission to the completion of PCI and a one-month follow-up period.

**Figure 1:**
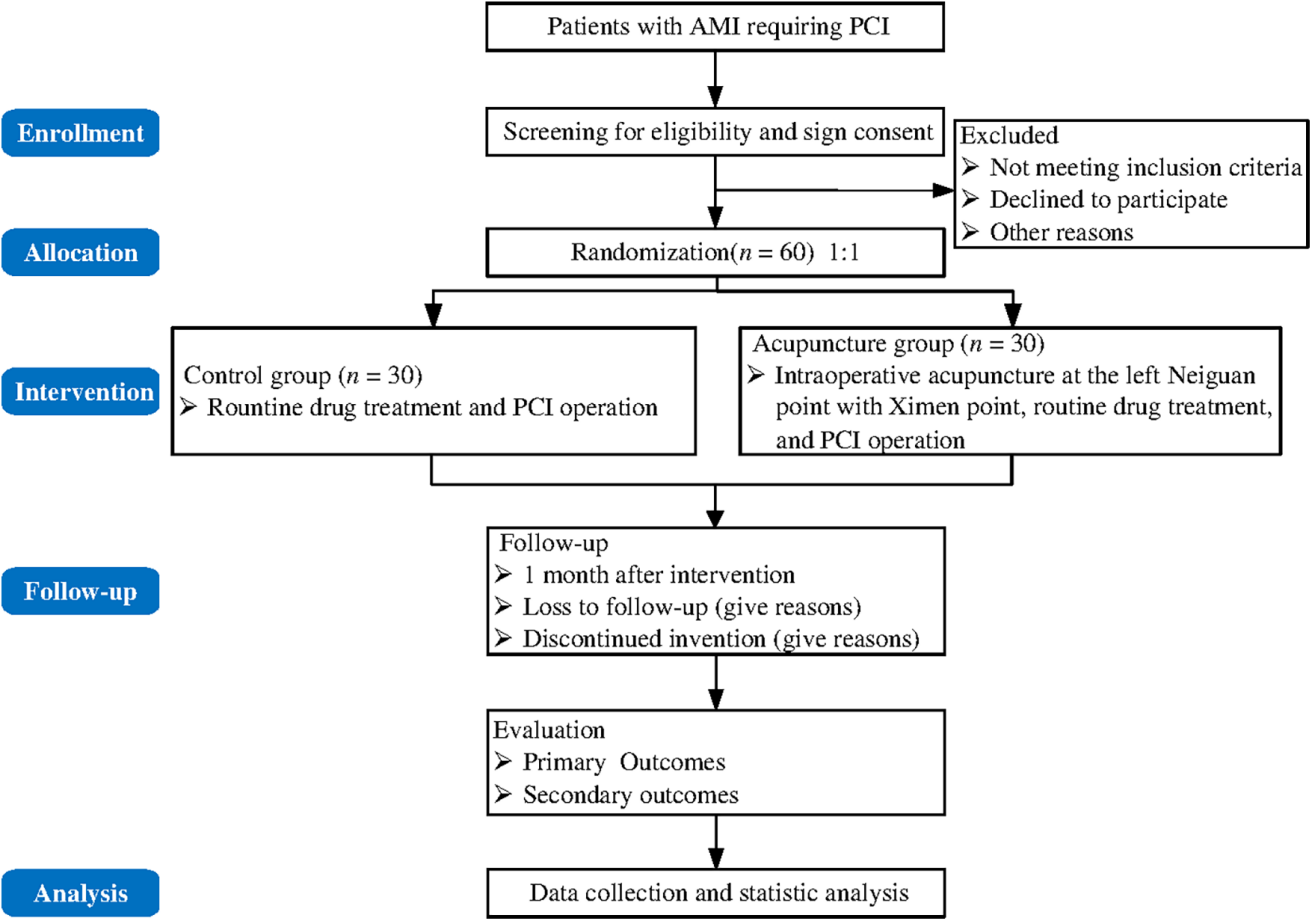
Flow chart of the trial.

The protocol will be designed in compliance with the Standard Protocol Items for Randomized Trials (SPIRIT). The SPIRIT checklist is included in the Supplementary Appendix. The trial flow chart is shown in Figure 1. The study design schedule is shown in Table 1.

**Table 1:** Study design schedule.

Study period	Treatment period			Follow-up period		
Timepoint	Preoperative PCI	Intraoperative PCI	Postoperative PCI	Postoperative		
				12 h	72 h	1 M
Demographic data^a^	√					
Routine drug treatment	√	** **	√	** **	** **	** **
Acupuncture (left Neiguan with Ximen)	** **	√	** **	** **	** **	** **
Incidence of Slow flow/no-reflow (TIMI and CTFC)	** **	√	** **	** **	** **	** **
Changes in cardiac biomarkers^b^	√	** **	** **	√	√	** **
Inflammatory markers^c^	√	** **	** **	√		** **
Pain score (NRS)	√	** **	√	√	** **	** **
VAS-A scores	√		√	√	** **	** **
Electrocardiogram	√	** **	√	√	** **	** **
TCM symptom score	√	** **	** **	** **	√	** **
MACCE	** **	** **	** **	** **	** **	√
Adverse events	√	√	√	√	√	√
Safety assessments^d^	√	** **	** **	** **	√	

^a^ Including age, gender, BMI, smoking history, medical history, and symptom duration.

^b^ Including cTnI, Myo, CK-MB, NT-proBNP.

^c^ Including leukocytes, neutrophils, lymphocytes, and Hs-CRP.

^d^ Including blood routine, electrolytes, liver and renal functions.

### 2.2 Participants and recruitment

The study is scheduled to be conducted from August 2023 to June 2025 at Yueyang Hospital affiliated with Shanghai University of Traditional Chinese Medicine. Sixty AMI patients requiring PCI will be recruited from the Emergency Department and undergo PCI in the cardiac catheterization laboratory. The diagnostic criteria for inclusion will be based on follow the Fourth Universal Definition of Myocardial Infarction (2018) (36). The investigator will describe the study details, including the purpose, benefits, and potential risks of the study to the patients. Patients agreeing to voluntarily participate in this study will need to provide signed informed consent, undergo eligibility assessment, and provide demographic information. The intervention and follow-up will adhere to the protocol. Participants will be allowed to withdraw without penalty.

### 2.3 Inclusion criteria

(1) Patient age between 18 and 80, regardless of gender.
(2) Patients meet the diagnostic criteria for AMI and schedule for PCI.
(3) Patients provide informed consent to participate.

### 2.4 Exclusion criteria

(1) Patients with severe liver, kidney, and hematopoietic system impairments (e.g., serum alanine aminotransferase >3 times the normal value or serum creatinine ≥265 μmol/L).
(2) Patients unable to cooperate or complete the PCI procedure because of alcoholism or cognitive dysfunction.
(3) Patients with severe cardiac complications, including cardiogenic shock.
(4) Patients unsuitable for acupuncture because of skin damage or infection.
(5) Patients who have undergone surgery in the last four weeks and have a risk of bleeding, during the procedure, or patients who are participating in other clinical trials.

### 2.5 Termination and dropout of trial patients

(1) Patients with poor protocol compliance and failing to receive treatment.
(2) Patients with incomplete medical records that impact evaluating the efficacy.
(3) Voluntary withdrawal by the patient.
(4) Adverse events (AEs) that render the participant unfit to continue.
(5) Patients considered as not suitable to continue by the researcher.

### 2.6 Randomization and allocation concealment

To avoid selection bias, an independent statistician, who will not be involved in any other part of the study, will randomly allocate eligible participants to the acupuncture group or the control group in a 1:1 ratio using the SPSS 25.0 software. Grouping will be performed based on stratified randomization, in which baseline variables such as gender, age, and presence of ST-segment elevation myocardial infarction will be used as baseline characteristics to balance the two groups. Opaque envelopes will be used to store the randomization scheme and will be opened successively according to the enrolment order. Grouping of patients will be determined according to the allocation scheme in the envelope. After opening the envelope, the assigned intervention will only be disclosed to the treating acupuncturist, whereas other researchers and the medical staff will remain blinded. Participants will receive treatment in separate rooms throughout the trial period to prevent communication.

### 2.7 Blinding

At recruitment, participants will be informed that they will have an equal chance of receiving EA during PCI. The acupuncturists and participants cannot be blinded because of the nature of the acupuncture intervention but steps will be taken to minimize bias resulting from single blinding as much as possible. Acupuncturists will receive specialized training before participating and will be prohibited from discussing the treatment procedures and outcomes and will not participate in the evaluation of results. Patients will be treated separately to prevent contact between participants. The randomization staff and acupuncturists will have access to the assignment information but the outcome evaluators and statisticians will remain blinded throughout the entire clinical trial. Statisticians will speculate on the grouping allocation before data analysis to assess the blinding efficacy and unblinding will only occur after completion of the data analysis.

Emergency unblinding will only occur in the event of severe AEs that would require immediate identification of the patient treatment, including details about severe infection, uncontrollable pain, unexplained death, or similar situations.

### 2.8 Interventions

#### 2.8.1 Control group (standard PCI treatment)

(1) *Fundamental nursing care*: This will include educating the patients regarding bed rest, avoiding overexertion, regulating diet, strictly avoiding smoking or alcohol consumption, monitoring vital signs, nutritional support, administering oxygen as appropriate, and timely management of the AMI-related complications.
(2) *PCI operation:* All patients will undergo PCI under local anesthesia after signing informed consent. Following standard coronary angiography, PCI will be performed by at least two experienced interventional cardiologists according to the 2021 ACC/AHA/SCAI Guideline for Coronary Artery Revascularization (37). The right radial artery approach will be preferred, but the femoral artery approach will be used in cases of contraindications. Intravenous heparin will be administered to maintain an activated clotting time (ACT) of above 250 s during the procedure. Stent selection and quantity will depend upon the vascular conditions and judgment of cardiologists. Thrombolysis in myocardial infarction (TIMI) flow grades and corrected thrombolysis in myocardial infarction frame count (CTFC) will be calculated post-stent implantation to assess the coronary artery blood flow and the incidence of SF-NR.
(3) *Routine drug treatment:* This will be according to the 2017 European Society of Cardiology (ESC) Guidelines for the Management of Acute Myocardial Infarction in Patients Presenting with ST-segment Elevation and the 2016 Guidelines for the Diagnosis and Treatment of non-ST-segment Elevation Acute Coronary Syndrome by the China Society of Cardiology of Chinese Medical Association (38, 39). Unless contraindicated, all patients will be administered 300 mg aspirin, 180 mg ticagrelor, and 40 mg atorvastatin before PCI. Postoperatively, patients will be prescribed secondary prevention medications, including aspirin (100 mg daily), clopidogrel (75 mg daily) or ticagrelor (90 mg twice daily), atorvastatin calcium tablets (20 mg nightly), angiotensin-converting enzyme inhibitors (ACEIs), angiotensin II receptor antagonists (ARBs), Beta-receptor blockers, and low-molecular heparin. The dose adjustment will be according to patient's condition.

#### 2.8.2 Acupuncture group (intraoperative acupuncture-assisted PCI treatment)

Patients in the acupuncture group will receive the same treatment as the control group. They will also receive intraoperative EA that will be administered to the AMI patients without interfering with the PCI procedure.

**Figure 2:**
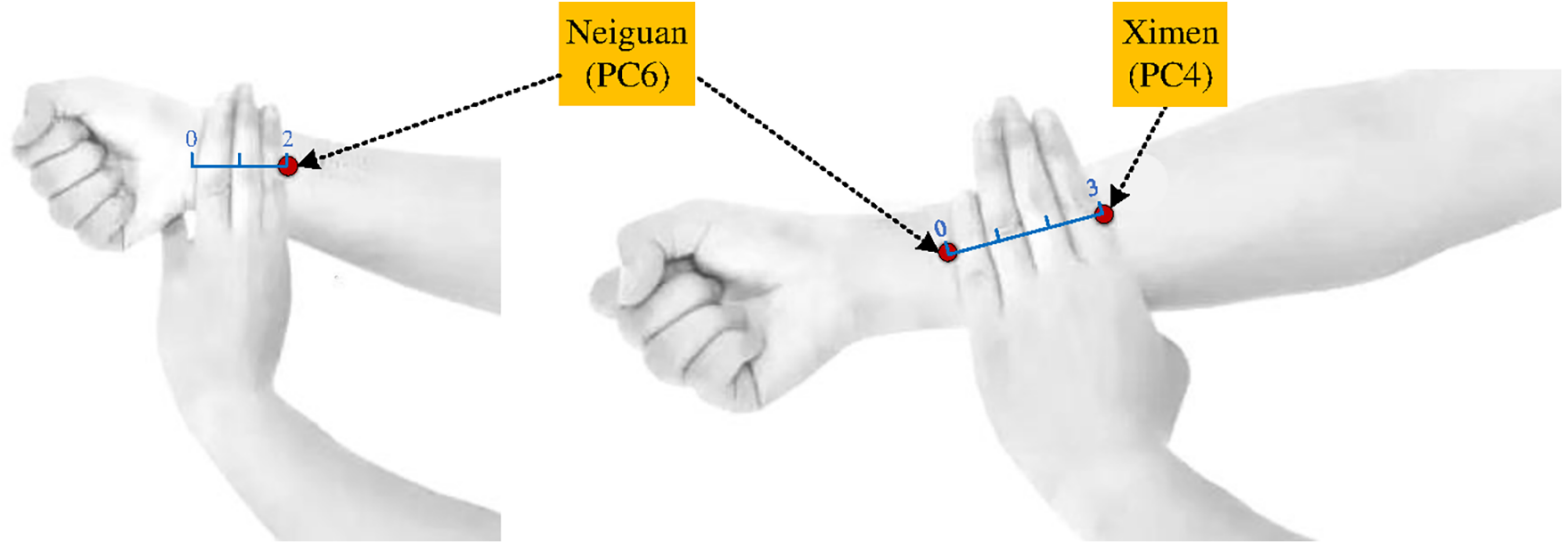
Location of acupoints.

(1) *Acupoints*: As shown in Figure 2, left Neiguan (PC6) and Ximen (PC4) were chosen as acupoint locations based on the Nomenclature and Location of Acupuncture Points (GB/T 12346-2006), a standard of the People's Republic of China
(2) *Implementation:* In the cardiac catheterization laboratory for PCI, the acupuncturist will sterilize the skin in the local area of the left Neiguan (PC6) and Ximen (PC4). Then, sterile disposable stainless steel acupuncture needles (1.5-inch Hua Tuo brand, *ϕ* 0.30 × 40 mm; Suzhou Medical Appliance Factory Ltd.) will be inserted vertically to a depth of 0.5–0.8 inch. Acupuncture manipulation, such as lifting and twisting, will be used until the De Qi sensation is felt. The EA therapeutic instrument (low-frequency electronic pulse therapy instrument, model G6805-2A, Shanghai Huayi Medical Instrument Co., Ltd.) will then be connected to enhance the acupuncture sensation based on the tolerance of the participant. Direct needling is the routine method for the PC6 and PC4 acupoints. Oblique needling is only used in cases requiring the penetrating needling technique. Therefore, this study will use the routine direct insertion method. Previous studies have demonstrated that continuous wave EA at a frequency of 20 Hz effectively increased blood flow in the ischemic area, reduced the infarct area, relaxed the muscles, relieved pain, decreased mortality, suppressed arrhythmias, and protected the myocardial tissue (40–43). Therefore, in this study, continuous wave EA stimulation at 20 Hz will be maintained throughout the PCI procedure and the current intensity will be adjusted based on the patient tolerance levels. Furthermore, experts will supervise and guide the operators throughout the experiment and promptly address any issues that may arise to ensure smooth progression of the surgeries. These measures will prevent any interference with the PCI procedure.

### 2.9 Outcome measurement

#### 2.9.1 Primary outcome

The primary outcome is the incidence of SF-NR and will be calculated as follows: Incidence of SF-NR = (number of cases with no-reflow + number of cases with slow flow)/total number of cases × 100%. SF-NR will be determined based on the CTFC and TIMI flow grade criteria. Two experienced cardiologists will independently assess slow flow (TIMI grade 2 or CTFC > 27 frames) and no-reflow (TIMI grade 0–1 or CTFC > 40 frames) immediately after PCI stent implantation for patients without dissection, thrombus, or spasm (44). The recordings will be conducted at a rate of 30 frames per second, and the CTFC of the left anterior descending artery will be calculated by dividing the number of frames by 1.7 (45). In the case of disagreements, data will be reassessed jointly to reach a consensus.

#### 2.9.2 Secondary outcomes

(1) *Cardiac biomarkers*: Assessments of serum high-sensitive cardiac troponin I (cTnI), myoglobin (Myo), creatine kinase-MB (CK-MB), and N-terminal pro-brain natriuretic peptide (NT-proBNP).
(2) *Inflammatory biomarkers*: Assessments of leukocytes, neutrophils, lymphocytes, and high-sensitivity C-reactive protein (Hs-CRP).
(3) *Chest pain score*: Chest pain severity will be assessed using the Numeric Rating Scale (NRS) with the participants rating pain on a 10-point scale, where 0 represents no pain, 1–3 indicates mild pain, 4–6 moderate pain, and 7–10 indicates severe pain.
(4) *Anxiety score*: This will be evaluated using the Visual Analogue Scale for Anxiety (VAS-A) with 10-point scoring system where 0 indicates no anxiety, 1–3 indicates mild anxiety, 4–6 indicates moderate anxiety, and 7–10 indicates severe anxiety.
(5) *Electrocardiogram examination*: Assessment of cardiac activity using the electrocardiograms.
(6) *TCM symptom score*: Symptom severity grading will be based on the 2002 Guidance principle of clinical research on new drugs of Traditional Chinese Medicine (46). The severity grading categories are none, mild, moderate, and severe. Major symptoms will be scored as 0, 3, 6, or 9 points. The secondary symptoms will be scored as 0, 1, 2, or 3 points. The specific scores are shown in the Supplementary Material. TCM symptom score = major symptoms score + secondary symptoms score. The efficacy index will be calculated using the following formula: [(Score at initial admission - Score at 24 h after PCI)/Score at initial admission] × 100%. The efficacy index is classified into the following three grades: ineffective (0%–30%), effective (30%–70%), and significant (70%–100%). Total effective rate = significant effective rate + effective rate.
(7) *MACCE*: The incidences of major adverse cardiovascular and cerebrovascular events (MACCE) include hemorrhage, recurrent myocardial infarction, revascularization, readmission, and cardiogenic death during the follow up within one month after PCI.

### 2.10 Safety assessments

All the participants will undergo laboratory tests, including routine blood tests, levels of electrolytes, as well as liver and renal functional tests immediately after admission and three days after surgery. Treatment safety will be assessed based on these test results. Participants will provide consent for these biological samples as an addendum when they sign the informed consent form.

### 2.11 Adverse events

Adverse events (AEs) are unexpected symptoms, signs, or health conditions associated with the treatment. Pre-existing health conditions or diseases will be categorized as AEs only if they worsen post-treatment. Abnormal laboratory tests will be considered as AEs if they represent clinically significant symptoms that require treatment. Common adverse reactions to electroacupuncture include broken needles, fainting, bleeding, bruising, infection, swelling, and other discomforts following needle insertion such as post-acupuncture pain, nausea, palpitations, dizziness, headache, and insomnia. Detailed AE data will describe the symptoms, severity, onset date, relationship with study manipulation, measures taken in response (including drug combination, dosage, start and end times), intervention methods (such as ceasing acupuncture), duration, follow-up, and outcomes. Follow-up will be conducted immediately after enrolment, and timely reports will be submitted to the principal investigator until all the issues are resolved. The investigator has the authority to determine trial termination and provide economic compensation for study-related harm. Loss of follow-up will also be recorded if the investigator cannot obtain information regarding adverse events.

Serious Adverse Events (e.g., resulting in death, hospitalization, or disability) will be promptly reported to the principal investigator and the Medical Ethics Committee. The Case Report Form (CRF) will document the time of death and autopsy results, if applicable.

### 2.12 Calculation of sample size

Since previous RCTs have not investigated acupuncture as an adjunct intervention for managing SF-NR in the AMI patients during PCI, it is challenging to determine the optimal sample size through power calculation. Therefore, instead of a formal sample size estimation, this trial will use a preliminary exploratory clinical pilot design for estimating the sample size (47). However, to preliminarily assess the feasibility of acupuncture-assisted PCI to prevent SF-NR, we will meticulously ensure adequate number of participants in our experimental design by adhering to the established guideline of a minimum of 12 participants per group for pilot studies (48). A previous study suggested that each group should have a sample size ranging from 20 to 25 cases for exploratory clinical trials assessing the effectiveness of intervention measures (49). Therefore, in this study, each group consisted of 25 patients and a total of 50 cases for the 2 groups. However, to account for a dropout rate of 20%, this study will recruit 60 patients (30 patients per group). It seems plausible that the dropout rate would be less than 20% because of the short duration of the procedure and the high requirement of surgery among the participants (50). The results of this study will contribute to providing the effect size data for calculating sample sizes in the further large-scale RCTs.

### 2.13 Data collection and management

An Electronic Data Capture (EDC) system will collect the clinical data of the participants. Two trained investigators will enter and reconcile data independently to maintain accuracy. They will complete the Case Report Form (CRF) for each participant. A research assistant will then verify the data accuracy and integrity, and address errors, omissions, or the need for clarification. A dedicated data administrator will oversee the system without data modification or access until participant enrolment, observation, and data collection are completed. In the CRFs, investigators will indicate changes by drawing lines, record modified data, as well as sign and date without erasing or overwriting original records. Both paper and electronic records will be stored for at least five years post-publication. Biospecimens will be ethically disposed after monitoring and in accordance with the participant rights and interests. Regular database backups will be implemented to prevent loss of data. To ensure participant confidentiality and prevent information leakage, personal details such as names, phone numbers, ID numbers, and medical history will be anonymous.

### 2.14 Quality control and assurance

The trial protocol has undergone rigorous review by the experts in acupuncture, cardiovascular medicine, statistics, and methodology. Investigators will be identified post-eligibility screening and a consistent staff will be maintained throughout the study. All the researchers, including acupuncturists, evaluators, and statisticians will receive pre-initiation training for consistently implementing the protocols. Regular meetings will be held to review the study progress, address any issues, and ensure protocol adherence. Laboratory tests will follow standard operating requirements with prompt explanations for any changes during the study. Licensed acupuncturists with over two years of experience will administer acupuncture treatment exclusively while avoiding other therapies such as acupressure and moxibustion. Furthermore, experts will supervise and guide the operators throughout the procedures, and promptly address any issues that may arise and ensure the smooth progression of surgeries. High patient compliance will be ensured because of the nature of the emergency surgery, and will be supported by drug counting, regular checks, compensation, and health education. The reasons for dropouts and withdrawals, if any, will be carefully documented. While improving patient compliance, researchers will strictly limit the inclusion criteria and use fully randomized, blinded, and concealed assignments. Efficacy and safety indicators will be evaluated and documented by independent assessors who are unaware of the group assignments. Medical records will be strictly confidential, with anonymous data processing. The data will be accessible only to the authorized researchers and the sponsor representatives. Throughout the trial, all the investigators will adhere to strict confidentiality policies.

### 2.15 Statistical analysis

Statistical analysis will be performed according to the intention-to-treat (ITT) principle using the SPSS V.25.0 software (IBM Corporation, Armonk, New York, USA). Missing data will be addressed through multiple imputations. *P* < 0.05 will be considered as a statistically significant difference between groups. The normally distributed data will be presented as Mean ± SEM. The inter-group comparisons for this data will be performed using the independent sample t-tests and paired t-tests will be used within-group comparisons. Non-normally distributed data will be presented as median (interquartile range) and the independent sample non-parametric tests will be performed for comparing the data between group. Count data will be expressed as frequency (percentage) and analyzed using the chi-square test. Ridit analysis or rank sum tests will be used to assess the ordinal data.

### 2.16 Patient and public involvement

Participants will not be part of the study design, conduct, outcome assessment, reporting, or dissemination. However, we will share our findings with the participants and the public through hospital social media, academic lectures, and peer-reviewed publications.

### 2.17 Ethics and dissemination

This study protocol will adhere to the Declaration of Helsinki guidelines. The Ethics Committee at the Yueyang Hospital has approved this study protocol (Approval No. 2023-090-01). Participants will provide written informed consent, affirming their right to withdraw at any time and emphasizing privacy and confidentiality. Any protocol modifications will require re-approval through a “Protocol Modification Specification.” Informed consent will be available online. The study findings will be disseminated through online peer-reviewed publications.

## 3 Discussion

Acupuncture shows the potential for improving microvascular perfusion in clinical studies but its efficacy in alleviating the SF-NR phenomenon in the AMI patients during PCI has not been established. A previous study reported the beneficial therapeutic effects of acupuncture in patients with slow flow after PCI (51). However, currently, there are no published reports regarding the role of acupuncture in preventing the complications from SF-NR during the PCI procedure. Therefore, we designed this trial to determine whether intraoperative electroacupuncture was effective in alleviating SF-NR in AMI patients undergoing PCI. AMI patients undergoing PCI alone were considered as controls. To the best of our knowledge, this study represents the first randomized controlled trial aimed at investigating the feasibility and clinical efficacy of intraoperative electroacupuncture-assisted PCI in preventing SF-NR in a diverse population of AMI patients.

Acupoint selection is crucial for the success of acupuncture treatment, with the primary principle being that the acupoints linked through the meridians. The pericardium meridian is generally used for the treatment of cardiovascular diseases (52). Based on previous literature and clinical experience, there is consensus among researchers for using PC6 and PC4 acupoints to treat patients with cardiovascular diseases. PC6 is the Luo-connecting point of the pericardium meridian and is traditionally used to treat heart-related conditions for alleviating circulation problems by regulating qi and activating the meridians to relieve pain (53, 54). PC4 is the cleft point of the pericardium meridian that is commonly used in acute and critical cases to invigorate Qi and blood, calm the heart, and soothe the spirit (55, 56). Stimulating these acupoints with “De Qi” sensation can regulate Qi and blood flow, enhance cardiovascular function, promote anti-inflammatory and antioxidant effects, and relieve pain and anxiety (57–59). Furthermore, to avoid interfering with the coronary stent implantation route, acupuncture will be performed on the left side. The selection of acupuncture points in this trial are consistent with the principles of “treatment based on syndrome differentiation” in TCM.

SF-NR is a common and serious PCI complication associated with poor prognosis. In this study, we will evaluate several secondary outcomes in addition to assessing the incidence of SF-NR as the primary outcome. We will measure the cardiac biomarkers to monitor the status of AMI (60). We will assess inflammatory biomarkers such as leukocytes, neutrophils, lymphocytes, and Hs-CRP, which are linked with SF-NR and are predictors of adverse cardiac events in the patients that have undergone PCI (61–63). Patient pain levels will be quantified using the NRS Chest Pain Scale, an internationally recognized method. Since long-term pain has significant emotional impact (64, 65), we will also use the VAS-A scale to determine if acupuncture alleviates or decreases emotional disorders in patients with AMI (66). The TCM symptom score will be used to assess the therapeutic effects of acupoint selection based on syndrome differentiation. Electrocardiography will be used to investigate the potential treatment effects of acupuncture in the patients with AMI. A one-month follow-up will assess the lasting effects of the treatment and the cardiovascular events. These comprehensive measures will thoroughly evaluate the effects of intraoperative acupuncture on various aspects of SF-NR. At the completion of the trial, we will evaluate recruitment time, participant consent rates, adverse events, blinding success, and feasibility of the outcome measurement tools. Furthermore, we will use flexible approaches for the follow-up, including remote video questionnaires, to minimize the dropout rates.

This study aims to confirm the efficacy of acupuncture as an adjunct to standard AMI therapy with strict quality control. However, there are few limitations. Firstly, conducting a fully randomized placebo-controlled acupuncture trial is challenging because of the nature of electroacupuncture intervention. To overcome this, we will implement a series of measures to minimize the single-blind bias as much as possible. We have meticulously designed our trial and adhering to methodological standards for allocation concealment and blinding will enhance the generalizability of our findings. To avoid unwarranted interference or risk to AMI patients through sham needling and focus more closely on studying the effects of acupuncture, this pilot study will use a non-acupuncture control group and implement blinding for the outcome evaluators and statisticians to minimize bias. Secondly, cardiac magnetic resonance (CMR) is considered the preferred method for assessing microvascular damage and offers benefits in the assessment of myocardial perfusion and cardiac function (67). In future clinical trials, the potential utilization of CMR for evaluating the cardioprotective effects of acupuncture will be explored. Thirdly, because of the financial and workforce constraints, this trial is not a multi-center study. This would result in a small sample size and therefore, the results may be potentially influenced by the geographical factors. Therefore, larger multi-center RCTs will be necessary to validate the results of this pilot trial. Finally, the pathophysiological mechanisms involved in coronary microvascular dysfunction, injury, and obstruction include microembolization, edema/capillary obstruction, RBC stasis, capillary rupture, leukocyte adhesion, and impaired vasomotion (68–70). However, the precise myocardial protective mechanisms of acupuncture remain ambiguous. We will intend to seek approval from the ethics committee to retain blood samples from patients both pre- and post-treatment, with the goal of investigating relevant plasma biomarkers to elucidate the therapeutic mechanisms of EA.

In summary, this pilot trial will investigate the role of electroacupuncture in preventing SF-NR during PCI and address the current research gaps. If proven effective, it will provide a new therapeutic strategy against SF-NR and lay the foundation for subsequent multicenter clinical trials. Furthermore, this trial will offer a new alternative for the clinical management of AMI by synergizing traditional acupuncture techniques with modern cardiovascular interventions for optimal patient care, prevent PCI-related complications, and guide treatment strategies for other severe cardiovascular diseases. This approach is more valuable when the conventional therapies are limited or contraindicated. It also promotes the application of TCM in modern medicine, establishes scientific credibility for TCM in new clinical areas, and provides a more comprehensive and effective choice for societal healthcare services. Future research should investigate the acupuncture mechanisms and the optimal timing of acupuncture treatment to further elucidate its potential benefits as a complementary therapy for patients with cardiovascular diseases.

## 4 Trial status

The trial recruitment is ongoing.

## Ethics statement

The studies involving humans were approved by the Ethics Committee of the Yueyang Hospital affiliated with the Shanghai University of Traditional Chinese Medicine (approval number: 2023-090-01). The studies were conducted in accordance with the local legislation and institutional requirements. The participants provided their written informed consent to participate in this study.
