# Supplementary material for: Electroacupuncture for slow flow/no-reflow phenomenon in patients with acute myocardial infarction undergoing percutaneous coronary intervention: protocol for a pilot randomized controlled trial
Source: Front Cardiovasc Med. 2024 Jun 18;11:1401269. doi: 10.3389/fcvm.2024.1401269 (PMC11218819; doi:10.3389/fcvm.2024.1401269)
Supplement: Supplementary file 1 [file Datasheet1.docx]

| **Table** TCM symptom score | | | | | |
| --- | --- | --- | --- | --- | --- |
| Quantification table of major symptoms | | | | | |
| Symptom | None  (0 point) | Mild  (3 point) | Moderate  (6 point) | Severe  (9 point) | |
| chest pain | Asymptomatic seizure | Attack up to 2 times a day, each time < 10 seconds, the pain is light, rarely need to take Quick-Acting Heart Reliever. | Attack 3-5 times a day, 10 to 30 seconds each time, more pain, more need to take Quick-Acting Heart Reliever | Attacks > 5 times a day, each lasting >30 seconds, affecting life, requiring frequent use of Quick-Acting Heart Reliever | |
| chest tightness | Asymptomatic seizure | Feel chest tightness after normal physical activity | Feel chest tightness  after mild activity | Feel chest tightness  when at rest | |
| Quantification table of secondary symptoms | | | | | |
| Symptom | None  (0point) | Mild  (1point) | Moderate  (2point) | | Severe  (3point) |
| shortness of breath | Asymptomatic seizure | Shortness of breath after general activity | Shortness of breath after slight activity | | Shortness of breath even when not active |
| palpitations | Asymptomatic seizure | occasional seizures with mild symptoms | frequent seizures but mild symptoms | | severe frequent seizures with severe symptoms |
